# Impact of Maternal COVID-19 Infection Versus Vaccination on Mucosal Immunity in Breastmilk

**DOI:** 10.3390/jcm15124494

**Published:** 2026-06-10

**Authors:** Mymy Nguyen, Rupsa C. Boelig, Julie Jones, Wathsala Wijayalath, Gregory D. Gromowski, Zubair H. Aghai, Elke S. Bergmann-Leitner

**Affiliations:** 1 Department of Obstetrics and Gynecology, Thomas Jefferson University Hospital, Philadelphia, PA 19107, USA; mymy.nguyen@jefferson.edu; 2 Sidney Kimmel Medical College, Thomas Jefferson University, Philadelphia, PA 19107, USA; julieajones66@gmail.com; 3 Agile Vaccines and Therapeutics Department, Naval Medical Research Command (NMRC), Silver Spring, MD 20910, USA; wathsala.k.wijayalatharachchige.ctr@health.mil; 4 Henry M Jackson Foundation for Advancement of Military Medicine, Inc., Bethesda, MD 20817, USA; 5 Viral Diseases Branch, Walter Reed Army Institute of Research, Silver Spring, MD 20910, USA; gregory.d.gromowski.civ@health.mil; 6 Nemours Children’s Health, Philadelphia, PA 19107, USA; zubair.aghai@nemours.org; 7 Biologics Research & Development, Walter Reed Army Institute of Research, Silver Spring, MD 20910, USA

**Keywords:** mucosal immunity, immune profiling, secretory IgA, antibody neutralizing activity

## Abstract

**Background/Objectives**: In the first months of their life, infants rely on maternal antibodies for immune protection. Breastmilk is a major source of these defenses, supplying secretory IgA, IgG, and IgM that help guard mucosal surfaces against pathogens such as SARS-CoV-2. Most studies on breastmilk immunity in the context of COVID-19 have emphasized circulating monomeric IgA, rather than the multimeric secretory IgA (sIgA) that is active at mucosal barriers. This study assessed in-depth the contribution of breastmilk antibody subtypes to SARS-CoV-2 neutralization capacity and how these profiles differ following maternal COVID-19 infection versus vaccination during pregnancy or postpartum. **Methods**: In this prospective cohort study, breastmilk samples were collected longitudinally from individuals who had COVID-19 during pregnancy or received COVID-19 mRNA vaccination during pregnancy or postpartum. Serological assays measured IgG, IgM, systemic IgA, and secretory IgA against SARS-CoV-2 spike and nucleocapsid antigens. **Results**: COVID-19 infection during pregnancy resulted in significantly higher systemic and secretory IgA levels compared to vaccination. Secretory IgA demonstrated a strong correlation with neutralization capacity. Principal component analysis revealed distinct antibody profiles in COVID-19-exposed individuals versus vaccinated cohorts, with significant overlap between pregnancy and postpartum vaccination groups. **Conclusions**: Although both COVID-19 vaccination and disease elicit sustained COVID-19-related antibodies in breastmilk, COVID-19 infection elicits a broader and more diverse antibody response in breastmilk, specifically with a greater secretory IgA generation. These findings support the value of maternal vaccination to safely confer mucosal immunity to neonates and the need for optimized vaccine formulations for mucosal immunity.

## 1. Introduction

The infant immune system relies heavily on passive immunity from the mother, especially in the first six months of life. Maternal immunoglobulins are transferred to infants through the placenta and breastmilk. Breastmilk antibodies aid in developing the neonatal microbiome, promote tolerization to allergens and commensal organisms, and—most importantly—provide protection against enteric and respiratory diseases [1]. Lower tract respiratory diseases continue to be a leading cause of infant hospitalizations, causing 14 million hospital admissions and 27,300 deaths between 2009 and 2019 in the United States [2]. However, exclusively breastfed infants have lower rates of respiratory infections, underscoring breastmilk’s significance in infant immunity to respiratory viruses [3].

Breastmilk antibody-mediated immunity depends on multiple immunoglobulin subtypes, predominantly secretory IgA, then IgG, and lastly secretory IgM [1]. Secretory IgA is responsible for providing passive mucosal immunity via three main mechanisms: direct pathogen neutralization, developing microbiota, and guiding healthy immune system maturation [4]. In the mucosa, secretory IgA has broad reactivity, making it ideal to be the first line of defense against a variety of antigens and microorganisms at the epithelium (reviewed in [5]). Secretory IgA is the most abundant in colostrum and declines over the first few months of life, thus highlighting the importance of exclusive breastfeeding in the first 6 months [4]. Alternatively, secretory IgG provides pathogen-specific immunity that directly neutralizes viruses or antigens that pass through the epithelial barrier [6]. IgG in breast milk is derived primarily from maternal serum and reflects both infection and vaccine-induced immunity [7]. Maternal IgG that is passed to the infant through breast milk triggers innate immune mechanisms, such as the complement pathway to eliminate foreign invaders [1]. The role of IgM is less well known but likely contributes to mucosal immunity like secretory IgA. IgM is present at lower concentrations than secretory IgA and function similarly as they can trap pathogens and promote microbiome colonization [1,8]. Notably, there is a compensatory increase in secretory IgM in individuals who are IgA deficient [9].

Breast milk immunity to respiratory viruses including SARS-CoV-2 arises not only from acquired infections, as levels of pathogen-specific IgA and IgG also rise in breastmilk after vaccination [10,11]. IgA levels in breast milk have been reported to emerge rapidly 1–2 weeks after vaccination and persist longer compared to IgG levels that require two doses of the vaccine [12]. Vaccination of mothers, therefore, remains the most important intervention to provide immunity to neonates, who are unable to mount an efficient humoral response [13].

Although it is well-established that maternal vaccinations contribute to breast milk immunity, it remains unclear how various antibody types contribute to neutralization capacity. The objective of this study was to evaluate how mucosal immunity contributes to neutralization capacity and varies following COVID-19 disease exposure vs. vaccination in the pregnant and postpartum periods.

## 2. Materials and Methods

### 2.1. Cohort Description

This is an analysis of breastmilk samples collected from participants who received either of the COVID-19 mRNA vaccines (the original formulation) during pregnancy (both doses), postpartum (at least one dose), compared to those who were exposed to COVID-19 during pregnancy in the time period from November 2019 to November 2021 as part of a prospective cohort study. Breastmilk was collected at delivery and 1, 2, and 3 months postpartum (or post first vaccine dose for those who received doses only postpartum).

### 2.2. Serological Assays

Breastmilk SARS-CoV-2 serology was assessed for IgM, IgG, secretory IgA (multimer), and systemic IgA (monomer) for nucleocapsid antigen-specific antibodies and spike antigen-specific antibodies. For assessing antibody specificity, the multiplex testing platform (MesoScale Diagnostics, Rockville, MD, USA) was used [14,15]. The antigens used were: Hemagglutinin (HA)-trimer from Influenza A (Hong Kong H3), spike (soluble ectodomain with T4 trimerization domain) trimers from SARS-CoV-2 (HU-1, P.1, B.1.1.7, B1.351), and the seasonal betacoronaviruses HKU-1 and OC43, as well as the spike N-terminal domain (NTD, Q14-L303 of the SARS-CoV-2 spike sequence), receptor binding domain (RBD, R319-F541 of the SARS-CoV-2 spike sequence), and nucleocapsid protein (N; full length) for SARS-CoV-2, and bovine serum albumin (BSA, as negative control). Assays were performed following the manufacturer’s instructions and as previously described [15]. Briefly, plates were blocked using Blocker A Solution and incubated at room temperature (RT) for 1 h on a plate shaker at 700 rpm. The plates were washed three times with 1x MSD Wash Buffer. Breastmilk samples were diluted to 1:100 with Diluent 100. The detection antibodies, SULFO-TAG anti-human IgG, anti-human systemic IgA, anti-human secretory IgA, or anti-human IgM antibody were diluted to 2 µg/mL in Diluent 100 (MSD). Secretory IgA—SULFO-TAG anti-human secretory IgA was generated as follows: sheep anti-human IgA polyclonal antibody specific to human IgA secretory component (sIgA) (MyBioscource, San Diego, CA, USA) was conjugated using MSD GOLD SULFO-TAG NHS-Ester Conjugation Pack (Mesoscale Diagnostics, Rockville, MD, USA) per manufacturer’s instructions. SULFO-TAG anti-human secretory IgA was used at 1 µg/mL final concentration.

### 2.3. Neutralization Capacity

The assay was performed as described previously [15]. The spike expression plasmid sequences for SARS-CoV-2 were codon optimized and modified to remove the last 18 amino acids of the cytoplasmic tail, which improves S protein-incorporation into pseudovirions (PSV). PSV were produced by cotransfection of HEK293T/17 cells with a SARS-CoV-2 S plasmid based on the Wuhan-Hu-1 genome sequence (GenBank accession number MN908947.3) and an HIV-1 pNL4-3 luciferase reporter plasmid (pNL4-3.Luc.R-E-, NIH HIV Reagent Program, catalog number 3418). S expression plasmids for SARS-CoV-2 VOCs were similarly codon optimized and modified, and included the following mutations: B.1.617.2/Delta (E156G, D614G, P681R, T19R, T478K, L452R, D950N, 157-158 del), B.1.1.529/Omicron (A67V, 69-70 del, T95I, G142D, 143-145 del, N211I, 212 del, G339D, S371L, S373P, S375F, S477N, T478K, E484A, Q493R, G496S, Q498R, N501Y, Y505H, T547K, D614G, H655Y, N679K, P681H, N764K, D796Y, N856K, N969K, Q954H, L981F). Infectivity and neutralizing titers were determined using ACE2-expressing HEK293 target cells (Integral Molecular, Philadelphia, PA, USA). Test sera were serially diluted, mixed with an equal volume of diluted PSV and plates were incubated for 1 h at 37 °C. Target cells were added to each well (40,000 cells per well) and plates were incubated for an additional 48 h. Relative light units were measured with the Synergy Neo2 Hybrid Multi-Mode Microplate Reader (Agilent BioTek, Winooski, VT, USA) using the Bright-Glo Luciferase Assay System (Promega, Madison, WI, USA). Neutralization dose–response curves were fitted by nonlinear regression using GraphPad Prism Version 10.2, and titers are reported as the reciprocal of the serum dilution necessary to achieve 50% inhibition of SARS-CoV-2 infectivity (ID50).

### 2.4. Statistical and Computational Analyses

*Data transformation and reporting.* Antibody quantification is reported as mean luminescence signal (MLS); all values were natural log transformed. Analyses were conducted using RStudio (Version 2026.01.0+392): *ggplot2*, *ggpubr*, *ggfortify* packages (for visualization), *corrplot*, *cluster* (for correlation matrix), *lfda*, *lme*, *lmerTest* for modeling. Significance was set at 0.05 for all analyses.

*Correlations.* Correlation matrices were generated to evaluate the relationship between antibody isotypes and neutralization capacity in each cohort and overall. All variables were standardized (z-score scaled) prior to analysis to enable comparability across different measurement scales. Data were stratified by cohort and pairwise Pearson correlations calculated. Correlation matrices were visualized using hierarchical clustering to group features with similar correlation patterns. Matrices containing non-finite values were excluded from visualization. This approach allowed identification of serological features that co-vary and potential associations with neutralization capacity.

*Principal component analysis (PCA)*: PCA was performed on scaled serological variables (i.e., SARS-CoV-2 spike- and nucleocapsid-specific IgA, sIgA, IgG, IgM, and neutralization titers). Missing values were imputed using variable-wise medians [16]. Samples were visualized with cohort-based coloring and 75% confidence ellipses, and variable loadings were overlaid using *ggfortify*.

*Linear Mixed-Effects Modeling.* Linear mixed-effects modeling was used to evaluate: (1) how visit (time), and nucleocapsid-specific IgG and IgM; (2) visit time, nucleocapsid-specific secretory IgA and systemic IgA; (3) spike-specific IgM and IgG; and (4) spike-specific secretory IgA and systemic IgA, were predictive of wild-type neutralization capacity. We applied linear mixed-effects models using the *lmer* function from the *lmerTest* R package. The dependent variable was the wild-type neutralization titer (ID50_WA1, log-transformed). Fixed effects included breastmilk antibody isotype (IgM, IgG, IgA, and sIgA) responses to SARS-CoV-2 antigens and the time point of sample collection (Visit: delivery, 30 days, 60 days, 180 days postpartum). Donor identity was included as a random intercept to account for repeated measurements. Models were fitted using restricted maximum likelihood (REML), and significance of fixed effects was evaluated by *t*-tests using Satterthwaite’s approximation for degrees of freedom. Model diagnostics were assessed through the inspection of residual and variance components.

*Principal component analysis (PCA)*. Unsupervised machine learning was applied to establish serological profiles for the different cohorts. Input for the PCA were all serological parameters. The R packages *lfda*, *ggfortify*, *ggpubr*, *and ggplot2* were used to generate the PCA plots. The weights of the individual parameters were indicated by the direction and the length of the vectors. The ellipses defining the various cohorts are based on a 75% confidence interval.

## 3. Results

### 3.1. Cohort Description

Baseline characteristics for the cohort are listed in Table 1. In the COVID-19 during-pregnancy (DPG) group, n = 8 (13.3%) with n = 5 with 12 samples (4%), no vaccine history and n = 3 with 15 samples (5%) that also received the COVID-19 vaccine in pregnancy. In the COVID-19 vaccine-in-pregnancy group (VPG), n = 29 (48.3%) with 143 samples (47.8%). In the COVID-19 vaccine-postpartum group (VPP), n = 23 (38.3%) with 129 samples (43.1%) of whom n = 4 received one dose in pregnancy and n = 19 received both doses postpartum. There were significant racial differences between groups and there was understandably a difference in trimester of vaccine receipt across groups. When comparing trimester of either vaccine received or COVID-19 diagnosis between those in the COVID-19 disease in pregnancy group and COVID-19 vaccine in pregnancy group, they were similar (*p* = 0.12, Kruskal–Wallis).

### 3.2. Longitudinal Change in CoV-2 Specific IgM and IgG in Breastmilk

**Figure 1:**
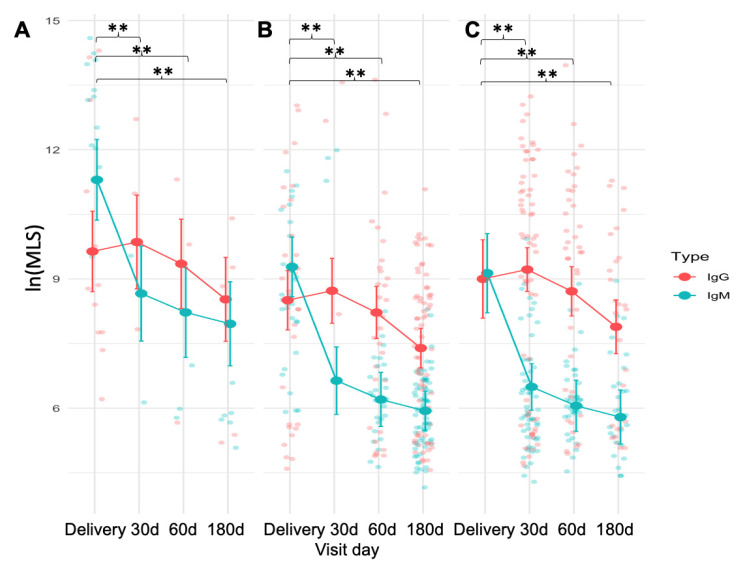
Longitudinal change in SARS-CoV-2–specific IgG and IgM antibodies in the different cohorts: (**A**) DPG cohort, (**B**) VPG cohort, and (**C**) VPP cohort. Line graphs represent group means (95% confidence interval), dots represent the individual samples. Total anti–SARS-CoV-2 antibody responses were calculated by summing serological measurements against SARS-CoV-2 spike, nucleocapsid, and N-terminal domain (NTD) antigens, and the sum was expressed as a natural log transformed mean luminescence signal (MLS). X axis indicates time point of sample collection (delivery, 30 days and 60 days postpartum). Faded points represent individual participants; solid lines indicate cohort means for each antibody type. Square brackets indicate statistically significant differences between time points for IgM responses (** adjusted *p* < 0.01). Changes in IgG responses failed to reach statistical significance.

The longitudinal analysis of SARS-CoV-2–specific IgM and IgG revealed distinct kinetics depending on the collection time point and the cohort (Figure 1). IgM antibody levels were highest at delivery for all three cohorts followed by a rapid decline. The differences between delivery and all subsequent time points were statistically highly significant (adjusted *p*-value < 0.01). Participants in the DPG cohort had the highest levels of IgM compared to the other two cohorts (*p* = 0.01 Kruskal–Wallis) (Figure 1A).

SARS-CoV-2–specific IgG levels demonstrated a slight increase after delivery in all cohorts 30 days postpartum. Later time points showed a gradual decrease in specific IgG levels. The VPP cohort showed the strongest increase in IgG levels compared to IgM, demonstrating response to the vaccine, but failed to reach statistical significance (adjusted *p* = 0.11) (Figure 1C).

### 3.3. Longitudinal Changes in CoV-2 Specific Systemic and Secretory IgA in Breastmilk

**Figure 2:**
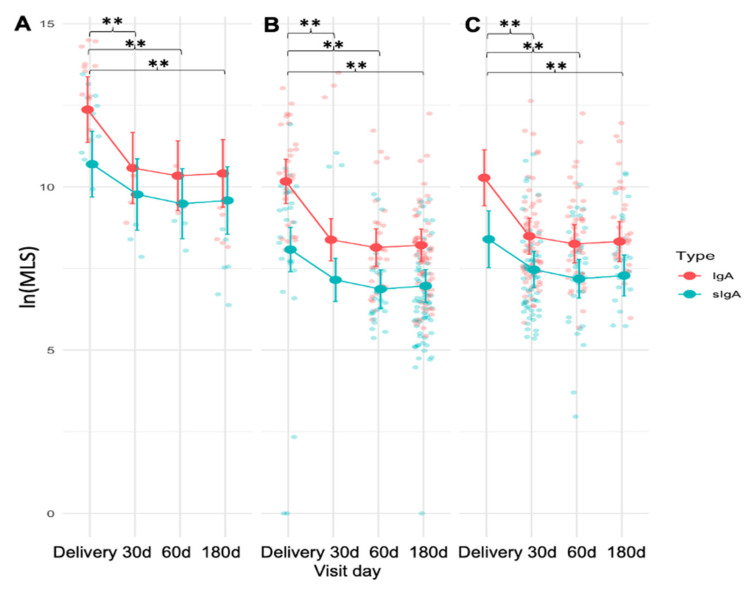
Longitudinal changes in SARS-CoV-2–specific secretory IgA (sIgA) and systemic IgA in the different cohorts: (**A**) DPG cohort, (**B**) VPG cohort, and (**C**) VPP cohort. Line graphs represent group means with 95% confidence intervals, and faded dots represent individual samples. Total anti–SARS-CoV-2 antibody responses were calculated by summing serological measurements against spike, nucleocapsid, and N-terminal domain (NTD) antigens and are expressed as natural log-transformed mean luminescence signal (ln[MLS]). Samples were collected at delivery and at 30, 60, and 180 days postpartum. Square brackets indicate statistically significant differences between time points for systemic IgA and sIgA responses (** adjusted *p* < 0.01; pairwise comparisons with Bonferroni correction).

The longitudinal analysis of SARS-CoV-2–specific IgA subclasses (systemic IgA and secretory IgA [sIgA]) revealed that antibody levels were highest at delivery and declined over time (Figure 2). For both systemic IgA and sIgA, antibody levels at delivery were significantly higher than those at 30, 60, and 180 days postpartum (adjusted *p* < 0.01). Despite this decline, detectable antibody responses persisted across all follow-up visits and remained relatively stable between 60 and 180 days postpartum. The DPG cohort exhibited the highest antibody titers compared to the VPG and VPP cohorts. Comparisons between systemic IgA and sIgA within the same time point demonstrated significant differences between the two antibody subclasses in the VPG and VPP cohorts at multiple time points. In contrast, in the DPG cohort, systemic IgA and sIgA differed significantly only at delivery (adjusted *p* < 0.001) and at 180 days postpartum (*p* = 0.03).

### 3.4. Cross-Variant and Seasonal CoV Antibody Breadth in Breastmilk

**Figure 3:**
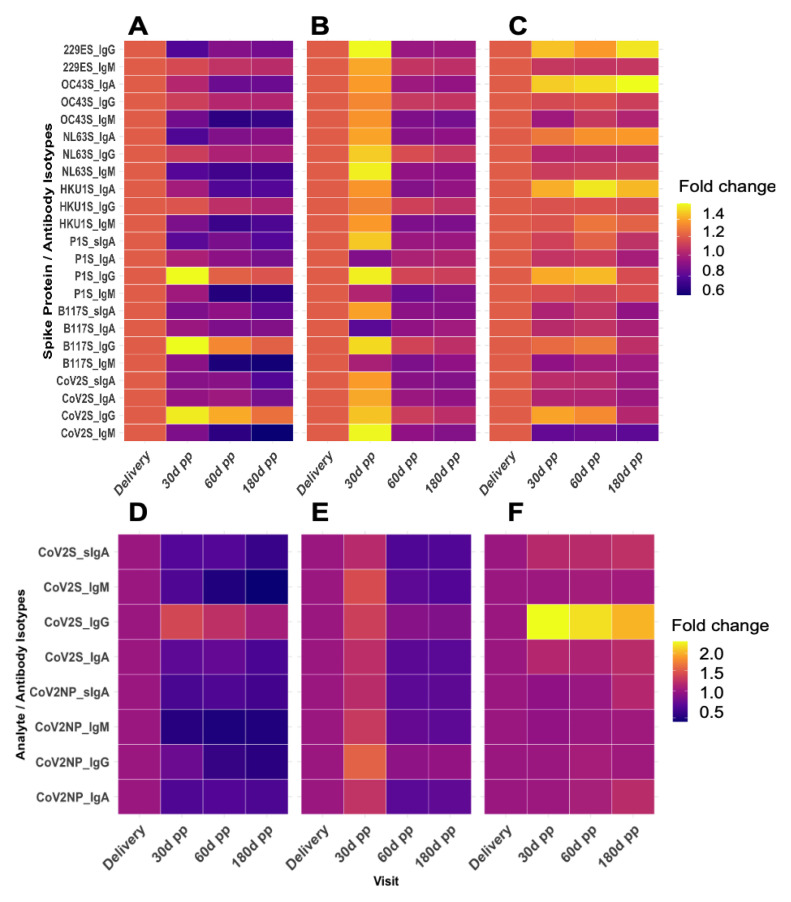
Longitudinal changes in the breadth of breastmilk antibody response. Heatmaps show median fold-changes in antibody titers relative to delivery (baseline) across postpartum visits (delivery, 30, 60, and 180 days) stratified by cohort: (**A**) DPG cohort, (**B**) VPG cohort, and (**C**) VPP cohort. Top panels depict the breadth of antibody responses to spike proteins from SARS-CoV-2, variants of concern (B.1.1.7, P.1, B.1.351), and seasonal human coronaviruses (HKU1, NL63, OC43, 229E). Responses are further stratified by isotype (IgM, IgG, IgA, and secretory IgA). Bottom panels show responses to SARS-CoV-2 spike and nucleocapsid proteins. Columns represent longitudinal time points. (**D**) DPG cohort, (**E**) VPG cohort, (**F**) VPP cohort. Color intensity indicates log-transformed fold-change relative to delivery. Positive values indicate increases and negative values indicate decreases relative to baseline.

To further define potential qualitative differences in the antibody breadth in breastmilk of mothers in the DPG cohort vs. the VPG or VPP cohorts, we tested breastmilk antibodies for their reactivity to spike proteins from the original SARS-CoV-2 virus (Wuhan Hu-1), variants of concern (B.1.1.7, P.1, B.1.351), and seasonal coronaviruses (HKU1, NL63, OC43, 229E) (Figure 3). Breastmilk antibody responses exhibited distinct longitudinal patterns across cohorts and antigens. At delivery, titers were relatively uniform across all CoV spike proteins and isotypes. Cohort DPG (Figure 3A) showed a general decrease in antibody levels over time across most antigens and isotypes. The only exceptions were IgG reactive to CoV2 Hu-1 and the VOCs on day 30 postpartum. In contrast, cohort VPG had a broader but not statistically significant increase at 30 days postpartum in CoV2-specific isotypes, particularly for IgM specific to SARS-CoV-2 (Wuhan Hu-1) and variant spike proteins (B.1.1.7 and P.1) as well as the seasonal 229E and NL63 spike proteins (Figure 3B). At later time points (60 and 180 days postpartum), antibody levels generally declined. Cohort VPP demonstrated a more sustained and moderate increase, with broader reactivity extending to seasonal coronaviruses, suggesting enhanced cross-reactivity across the various CoVs (Figure 3C). Pan-CoV-reactive secretory IgA responses followed similar trends but were generally lower in magnitude and more transient. Overall, these data indicate cohort-specific differences in the magnitude, durability, and breadth of breastmilk antibody responses, with the strongest and most transient boosting observed in cohort VPG.

Assessing the temporal changes in the antibody profiles revealed that antibody levels against both spike and nucleocapsid are declining at the same rate. The only exception is in the VPP where spike-specific IgG, (but not other isotypes) was significantly increasing, reflecting the effect of vaccination (Figure 3F, *p* = 0.03, pairwise comparison).

### 3.5. Neutralizing Antibody Titers in Breastmilk Associated with Prior COVID-19 Infection

**Figure 4:**
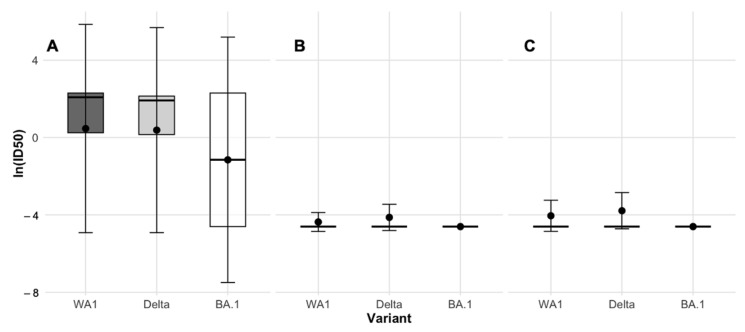
Neutralization titers across SARS-CoV-2 variants in different cohorts. ID50 neutralization titers are expressed as natural log-transformed values. Data are shown for three cohorts: (**A**) DPG cohort, (**B**) VPG cohort, and (**C**) VPP cohort. Boxplot represents the interquartile range with the median indicated by the black point, whiskers show the 95% confidence interval. Titers were measured against three SARS-CoV-2 variants: WA1, Delta, and BA.1. Statistically significant differences between cohorts were observed (Kruskal–Wallis test, *p* < 0.001).

Analysis of neutralization titers revealed that the DPG cohort displayed significantly higher neutralizing activity against WA1 and Delta variants, with slightly reduced titers against BA.1 (Figure 4). The differences in the neutralizing activities in the DPG cohort were significantly higher compared to the other two cohorts (*p* < 0.001, Kruskal–Wallis). In contrast, vaccinated cohorts (VPG and VPP) demonstrated minimal neutralization, with ID50 values near the assay baseline across all variants. These findings indicate that prior infection drives stronger neutralizing responses than vaccination alone in this dataset and highlight a potential reduction in activity against the BA.1 variant.

### 3.6. Establishing Cohort-Specific Serological Profiles

**Figure 5:**
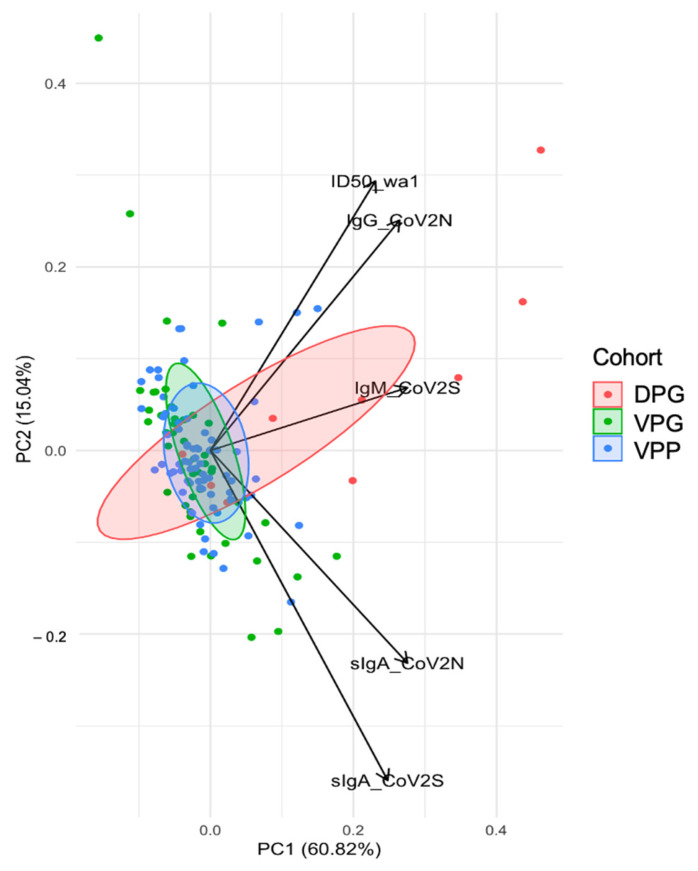
SARS-CoV-2–specific serological profiles in breastmilk. Principal Component Analysis (PCA) plots were generated by integrating all serological measures (specificity of antibodies, isotypes, neutralizing activity). Loading vectors indicate the direction and weight of each parameter (indicated by the length of the vector) to the serological profile of each group. Ellipses correspond to the 75% confidence interval for each group.

Next, we integrated all parameters (i.e., fine specificity to SARS-CoV-2 antigens, antibody isotypes and subclasses, neutralizing activity) to establish serological profiles for each cohort and identify potential overlaps using unsupervised machine learning (Figure 5). The principal component analysis (PCA) plot revealed a significant overlap between the vaccination cohorts (VPG and VPP). The profile of the DPG cohort revealed a far wider profile, differentiated primarily by SARS-CoV-2 nucleocapsid-specific IgG and secretory IgA as well as SARS-CoV-2 spike-specific IgM and secretory IgA and neutralizing activity to the SARS-CoV-2 Wa1 strain. Consistent with the fact that the COVID-19 vaccine only encoded the spike protein, COVID-19 infection results in a broader immune response compared to a vaccine that only induces antibodies to the spike protein. The cohorts receiving the vaccine during or after delivery were indistinguishable from each other in terms of their SARS-CoV-2 antibody profiles.

### 3.7. Correlations Between Systemic vs. Secretory IgA in Breastmilk and Neutralization

**Figure 6:**
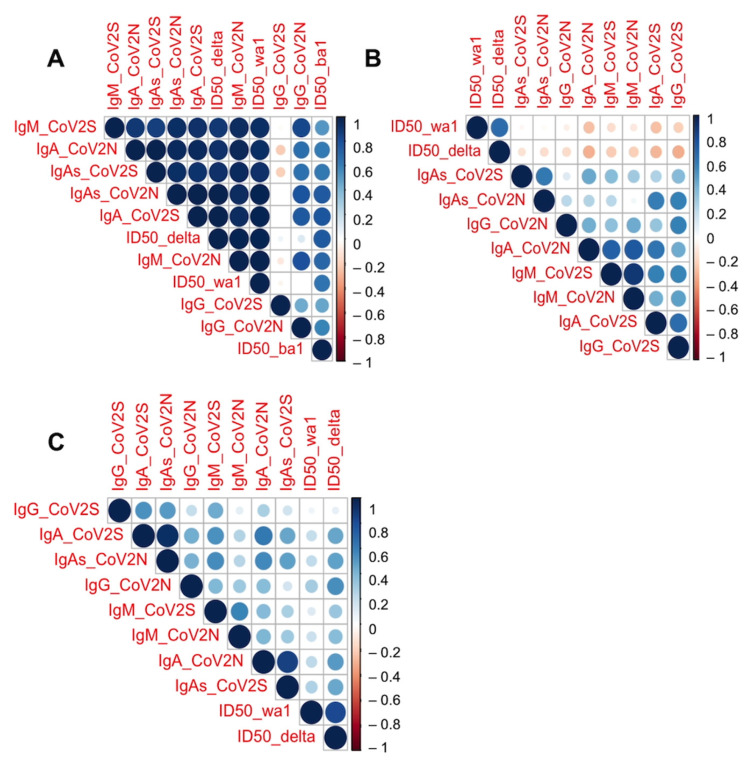
Correlation of IgA subtypes and Neutralization Capacity stratified by cohort. Panel (**A**) DPG cohort, Panel (**B**) VPG, and Panel (**C**) VPP cohort. The color and size of the circles within the correlation matrix correspond to pairwise Spearman correlation coefficients. The factors in the individual matrices are ordered according to the degree of association between variables.

After constructing integrated serological profiles across cohorts, we next sought to resolve the relationships between SARS-CoV-2–specific antibody features and functional antiviral activity. Correlation matrices were established to examine relationships between SARS-CoV-2–specific IgA subclasses and strain-specific neutralization activities, revealing cohort-specific patterns (Figure 6).

In the DPG cohort, strong positive associations were observed between IgA subclasses, IgM, and neutralization activity against the Wa-1 and Delta SARS-CoV-2 strains (Figure 6A), indicating that mucosal antibody responses were closely linked to functional neutralizing capacity in this group.

In the VPG cohort, the correlation matrix revealed a distinct relationship between spike-specific antibodies correlated with nucleocapsid-specific antibodies, suggesting that a subset of participants likely experienced a prior, unreported SARS-CoV-2 infection. The absence of correlations between SARS-CoV-2–specific IgA—both systemic and secretory—and neutralization activity was unexpected. This finding suggests that vaccination alone may not strongly induce IgA responses associated with neutralizing activity, and that infection-induced IgA-mediated neutralization may not persist over time. In contrast, the VPG cohort (Figure 6B) exhibited a more heterogeneous correlation structure, characterized by weaker associations and negative correlations between binding antibodies and neutralization, suggesting reduced coordination between humoral parameters. The VPP cohort (Figure 6C) demonstrated an intermediate profile, with moderate positive correlations and clearer segregation by antigen specificity and isotype. Notably, neutralization titers showed the strongest associations with SARS-CoV-2–specific IgA. Significant neutralizing activity was detected against both the WA-1 and Delta strains and demonstrated significant correlation with each other across all cohorts (*p* < 0.05). In contrast, none of the cohorts exhibited significant neutralization against the subsequently emerging Omicron variant, and therefore could not be included in the correlation matrices.

### 3.8. Identification of Serological Markers Associated with Viral Neutralization

**Figure 7:**
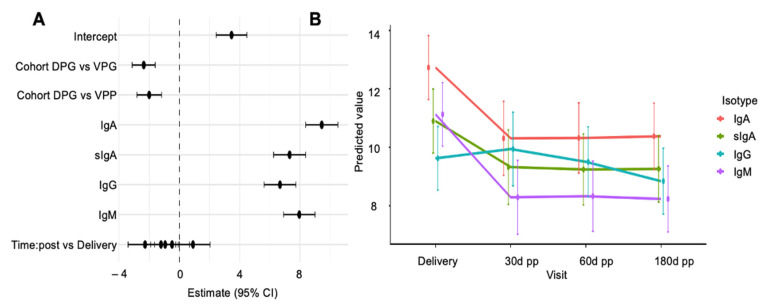
Modeling of antibody responses and their association with neutralization activity. (**A**) Forest plot of fixed effects estimates from a linear mixed-effects model. Coefficients with 95% confidence intervals are shown for Visit (Delivery vs. Post-Delivery), antibody Type (IgG, IgA, IgM, and secretory IgA), and Cohort (VPG and VPP, with DPG as the reference), including a random intercept for donor. Points indicate estimates and horizontal bars represent 95% confidence intervals; the dashed vertical line denotes the estimate = 0. (**B**) Predicted antibody levels for each isotype across study visits (Delivery, 30, 60, and 180 days postpartum), derived from the same model. Points represent estimated marginal means with 95% confidence intervals, and lines connect predictions across visits. Values are shown on the log-transformed scale of the outcome variable.

Building on PCA and correlation analyses, we employed multivariate linear mixed-effects modeling to validate and quantify the observed associations. By integrating breastmilk antibody isotypes/subclasses with antigen-specific responses, we identified coordinated serological signatures linked to neutralization capacity. Linear mixed-effects modeling identified antibody isotype as the primary determinant of response magnitude (Figure 7A). In the model, all isotypes (IgG, IgA, IgM, and secretory IgA) were significantly associated with neutralization, with IgA showing the largest effect size. In contrast, neither the cohort nor the time point of sampling had a significant effect in this model. Plotting temporal changes in the predicted values of the respective antibody isotypes revealed a general decline in antibody levels from delivery to postpartum time points (Figure 7B). According to the model, the most pronounced decrease in predictive value was observed for IgM, while IgG and sIgA remained relatively stable over time. Further dissecting the role of fine-specificity of antibodies in this model revealed that breastmilk IgM directed against SARS-CoV-2 spike protein and IgG directed against SARS-CoV-2 nucleocapsid emerged as the strongest predictors of wild-type (Wa-1) neutralization titers (both *p* < 0.001, Satterthwaite’s approximation), highlighting a previously underappreciated contribution of early and non-neutralizing humoral components to functional immunity. The model also indicated that nucleocapsid- and spike-specific secretory sIgA had strong associations with neutralization (*p* = 0.07 and *p* = 0.05, respectively, Satterthwaite’s approximation). Notably, the robust correlation between nucleocapsid-specific antibodies and neutralization, despite their inability to directly mediate viral neutralization, may position these responses as surrogate markers of protective immunity.

## 4. Discussion

The present study provides an in-depth characterization of serological responses in breastmilk induced by vaccination compared with COVID-19 infection. While a substantial body of literature has examined differences in antibody responses following mRNA vaccination and infection, comparatively little is known about how these exposures shape SARS-CoV-2–specific antibody subclasses and isotypes in mucosal compartments. Our study addresses this gap by directly comparing the composition of SARS-CoV-2–specific antibody isotypes and viral neutralizing activity in breastmilk from women with COVID-19 during pregnancy and those vaccinated either during pregnancy or postpartum. This study evaluated breastmilk antibody composition and in vitro neutralization capacity as a surrogate marker for protection but did not assess infant infection rates or clinical outcomes; therefore, conclusions regarding infant protection remain inferential.

Consistent with prior reports, we observed a robust transfer of IgA and IgM into breastmilk following infection, whereas vaccination was associated with detectable but substantially lower secretory IgA responses compared with natural infection [17,18]. To our knowledge, this study is among the first to systematically evaluate SARS-CoV-2–specific secretory IgA in breastmilk alongside longitudinal changes in antibody composition from delivery through 180 days postpartum (Figure 2) and their relationship to in vitro viral neutralization (Figure 6). Secretory IgA is a central mediator of mucosal immunity, uniquely adapted to protect epithelial surfaces, having significantly higher neutralizing potency than its systemic counterparts [19]. As a dimeric antibody complexed with the secretory component, secretory IgA exhibits enhanced avidity, resistance to proteolytic degradation, and the ability to agglutinate viral particles and prevent epithelial attachment [4,20]. These properties enable immune exclusion at mucosal barriers and support a potential role for secretory IgA in passive mucosal immune transfer through breastfeeding [4,21,22]. Previous studies in respiratory and enteric infections, including RSV, influenza, cholera, and rotavirus, have associated breastmilk-derived secretory IgA with reduced infant susceptibility to infection and disease severity [23,24,25].

At delivery, women with prior COVID-19 during pregnancy (DPG) exhibited the highest levels of SARS-CoV-2–specific IgM, IgG, IgA, and secretory IgA in breastmilk compared with vaccinated cohorts (VPG and VPP), mirroring what has been reported for systemic antibody responses [18,26,27]. Following delivery, all antigen-specific isotypes except IgG declined across cohorts, highlighting a dynamic and time-dependent evolution of the breastmilk antibody repertoire. This temporal evolution may reflect the transition from acute plasmablast-driven responses toward a more stable long-term antibody repertoire during the postpartum period. While previous studies have described early postpartum antibody composition [17] or short-term persistence [11,27] our data extend these observations by providing a longitudinal view and a more detailed isotype-specific analysis (Figure 1, Figure 2 and Figure 3).

Importantly, functional analyses revealed that the capacity to neutralize SARS-CoV-2 in breastmilk correlates with distinct antibody isotypes (Figure 4, Figure 5 and Figure 6): Early after delivery, spike-specific IgM showed the strongest association with neutralizing activity, whereas at later time points, nucleocapsid-specific IgG, unexpectedly, showed the strongest association with neutralization. Although nucleocapsid-specific antibodies are not directly neutralizing SARS-CoV-2 viral particles in vivo, their presence reflects prior infection and a broader, coordinated humoral immune response. Indeed, nucleocapsid-specific IgG has been implicated in Fc-mediated effector functions, including antibody-dependent cellular cytotoxicity and complement activation, which have been described in prior studies and may contribute to viral control through clearance of infected cells [28,29].

Notably, secretory IgA demonstrated a stronger association with neutralizing capacity than systemic IgA, underscoring the functional importance of mucosal antibody responses. This finding highlights the unique role of secretory IgA as the dominant antiviral immunoglobulin at mucosal surfaces and supports its potential relevance in mediating passive mucosal immune transfer to the infant. Importantly, secretory IgA (sIgA) and systemic monomeric IgA also differ fundamentally in their functional properties and anatomical origin, with sIgA being locally produced at mucosal sites and specifically adapted for immune exclusion at epithelial surfaces (reviewed in [30]). Effective protection at the respiratory epithelium, therefore, depends on the presence of locally induced mucosal immune responses, rather than solely on circulating systemic antibodies [31]. The observed association between nucleocapsid-specific IgA and neutralization likely reflects a highly polyfunctional antibody response, in which nucleocapsid reactivity serves as a proxy for the magnitude and quality of the broader antibody repertoire targeting neutralizing epitopes.

These findings reflect how SARS-CoV-2 exposure and vaccination influence the antibody landscape in breastmilk and provide insight into the potential transfer of SARS-CoV-2–specific mucosal antibodies through breastfeeding. Firstly, breastmilk delivers passive immunity with an evolving transition from IgM to IgG and secretory IgA, which offer varying levels of protection. Secondly, neutralization is associated with specific antibody subtypes, specifically IgM against spike protein and IgG against nucleocapsid. While nucleocapsid-specific IgG do not directly mediate neutralization, there are several reports that indicate the potential role of nucleocapsid-specific antibodies in the defense against SARS-CoV-2 [32]. The nucleocapsid protein is not only critical for the replication of the virus inside the mammalian host cell, but also plays a dominant role in the regulation of innate immune responses and viral pathogenesis [33,34]. It is therefore plausible that high levels of nucleocapsid-specific antibodies may reflect broader antiviral immune response, although direct protective effects were not assessed in this study. In case of nucleocapsid-specific IgG, the antibodies have been shown to mediate antibody-dependent cytotoxicity (ADCC) [28] and complement activation [29] facilitating immune clearance of infected cells. These observations support the interpretation that nucleocapsid-specific antibodies may serve as a surrogate marker of a broader protective immune response.

Identifying contributors to neutralization capacity is key to understanding the trajectory of the humoral response. Initially, IgM levels to the spike protein rise, indicating recognition of the antigen on the surface of the viral envelope, thus preventing viral entry into the host cell [35]. Later, IgG to nucleocapsid increases, reflecting a delayed but more specific response to the internal phosphoprotein responsible for viral RNA replication [36]. Lastly, IgA showes stronger neutralization capacity due to its dimeric structure and, thus, higher antigen avidity [37].

Comparing direct neutralizing activities against the viral particles mediated by SARS-CoV-2 spike antibodies revealed that IgA (systemic and secretory) had a stronger neutralization capacity than IgM and IgG, underscoring IgA’s dominant role in mucosal defense. Lastly, while those with prior COVID-19 infection have a more diverse antibody response and wider neutralization capacity, the infection, however, poses a significant risk to both mother and baby [38,39]. Vaccines were associated with a vaccine antigen-targeted and consistent response of antibody subtypes which is associated with reduced risk of infection and severe disease. Our data show that despite their shortcomings, mRNA-based, parenterally delivered COVID-19 vaccines were associated with detectable SARS-CoV-2–specific secretory IgA responses in breastmilk, although these responses were substantially lower than those observed following natural infection, supporting the concept that vaccinating pregnant women against SARS-CoV-2 may contribute to the transfer of SARS-CoV-2–specific mucosal antibodies to the newborn through breastfeeding. The lower levels of secreted IgA induced by vaccination versus infection indicates limited mucosal immune priming by parenteral vaccines. Optimized next-generation vaccine strategies, including mucosal or intranasal vaccine approaches, are needed to enhance mucosal immune priming and promote stronger secretory IgA responses. Improving mucosal immunity may also include the use of novel adjuvants that promote mucosal immunity even when the vaccine is delivered parenterally, such as dmLT [40]. Nevertheless, our findings also underscore the need to further refine vaccine design to better emulate the broad immune response developed from SARS-CoV-2 exposure and boost mucosal immunity.

The broader antibody profile following natural infection is not surprising given the distinct immunological environments associated with respiratory mucosal infection versus parenteral vaccination. SARS-CoV-2 infection exposes the mucosal immune system to prolonged antigenic stimulation across multiple viral proteins, including spike and non-spike antigens such as nucleocapsid, within the respiratory tract. This prolonged exposure is expected to contribute to broader germinal center reactions, affinity maturation, and clonal diversification, and establishment of mucosal memory B cell responses (refs). The compartmentalized mucosal imprinting is thought to play an important role in sustained sIgA production at sites of viral entry. In addition, the mucosal immune response to SARS-CoV-2 may prime virus-specific lymphocytes for homing to—and residence in mucosal tissues [41,42]. In contrast, intramuscular mRNA vaccination primarily induces a systemic immune response focused on the SARS-CoV-2 spike antigen, thus limiting the breadth of mucosal antibody diversification. Moreover, lymphocytes induced by parenteral vaccination may exhibit reduced homing to—or residency within—mucosal tissues, potentially limiting the generation of robust local mucosal immunity (reviewed in [43,44]). Consistent with this concept, previous studies have demonstrated enhanced mucosal antibody breadth and cross-reactive immunity following prior infection or hybrid immunity compared with vaccination alone [45,46].

Collectively, these findings support the concept that the anatomical site and context of antigen exposure are major determinants of the magnitude, breadth, and compartmentalization of breastmilk mucosal antibody responses. Next-generation vaccines against respiratory pathogens should induce stronger mucosal immune responses, particularly secretory IgA, in addition to robust systemic immunity [47]. Such vaccines will likely require mucosal delivery strategies, such as intranasal administration, to effectively induce local respiratory tract immunity and robust antigen-specific sIgA responses capable of blocking viral entry at the epithelial surface.

This study has several limitations that should be considered when interpreting the findings. First, the DPG cohort size was limited (n = 8), which may reduce statistical power and limit generalizability. In addition, cohort heterogeneity, including differences in timing of infection or vaccination relative to pregnancy and delivery, may have contributed to variability in antibody responses. Although participants in the vaccinated cohorts did not report prior COVID-19 infection, asymptomatic or unrecognized SARS-CoV-2 exposure cannot be fully excluded. Furthermore, the present study evaluated antibody composition and in vitro neutralization activity but did not assess infant clinical outcomes or directly measure Fc-mediated effector functions, or other mechanistic correlates of protection. Future studies incorporating larger cohorts and infant outcome data will be important to further define the relationship between maternal mucosal immunity and passive immune protection through breastfeeding.

From a translational perspective, the findings of the present study reinforce the limitation of parenteral vaccination in eliciting strong mucosal immune responses, despite their effectiveness in inducing systemic IgG-mediated protection. In contrast, mucosal vaccination strategies, including intranasal vaccines, have the potential to induce both systemic immunity and local secretory IgA responses at the respiratory epithelium, thereby providing a first line of defense against viral entry [48,49]. Such locally induced mucosal immunity may also influence the quality and magnitude of breastmilk-derived sIgA through the entero-mammary link, thereby improving passive immune protection in breastfed infants [26,50]. Several next-generation vaccine platforms are currently being evaluated for their ability to induce durable mucosal immunity and may represent an important advance over current intramuscular formulations for respiratory pathogens such as SARS-CoV-2.

## 5. Conclusions

Although both COVID-19 vaccination and disease elicit sustained SARS-CoV-2–specific antibodies in breastmilk, COVID-19 infection was associated with a broader and more diverse antibody response in breastmilk, particularly with respect to IgA and secretory IgA responses, specifically with a greater IgA generation. These findings support the value of maternal vaccination as a safe strategy to promote transfer of SARS-CoV-2–specific antibodies through breastmilk and highlight the need to optimize vaccine development through enhanced elicitation of mucosal immune responses.

## Figures and Tables

**Table 1:** Cohort Characteristics and Classifications.

Baseline Characteristics	
Age	33.5 ± 3.5
Body mass index (BMI)	25.1 ± 5.0
COVID disease in pregnancy	n = 8 (13.3%)
Vaccine in pregnancy, no COVID history	n = 29 (48.3%)
COVID vaccine completed post-partum (at least one dose postpartum)	n = 23 (38.4%) *n = 10 received one dose in pregnancy* *n = 13 received both doses postpartum*
Race	Black 4 (7%) White 50 (83%) Asian 4 (7%) Missing 2 (3%)
Hispanic Ethnicity	1 (1.7%)

## Data Availability

All data are contained within the manuscript. R scripts (Version 2026.01.0+392) can be obtained from the contributing author upon request.

## Abbreviations

The following abbreviations are used in this manuscript:

ADCC	Antibody-dependent cellular cytotoxicity
BMI	Body mass index
BSA	Bovine serum albumin
CoV2N	CoV2 nucleocapsid protein
COVID-19	Coronavirus disease of 2019
DPG	Disease during pregnancy cohort
h	Hour
HA	Hemagglutinin
HIV	Human Immunodeficiency virus
ID50	50% infective dose
Ig	Immunoglobulin
MLS	Mean luminescence signal
mRNA	messenger RNA
NTD	N-terminal domain
PCA	Principal component analysis
PSV	Pseudovirion
RBD	Receptor-binding domain
REML	Restricted maximum likelihood
RNA	ribonucleic acid
rpm	Rounds per minute
S	Spike protein
SARS-CoV-2	Severe acute respiratory syndrome coronavirus-2
sIgA	secretory IgA
VOC	Variant of concern
VPG	Vaccine during pregnancy cohort
VPP	Vaccine post-partum cohort
